# Significance of Interviral Recombination as Novel Mechanism for Extending Viral Disease Repertoire

**DOI:** 10.4172/2168-975X.1000217

**Published:** 2016-06-23

**Authors:** Edward M Johnson, Dianne C Daniel

**Affiliations:** Department of Microbiology and Molecular Cell Biology, Eastern Virginia Medical School, Norfolk, VA 23507, USA

**Keywords:** Progressive multifocal leukoencephalopathy, PML, Multiple sclerosis, MS, DNA replication, DNA recombination, Epstein-Barr virus, JC virus, HERV, DNA break-induced replication

## Abstract

The recent observation of interviral recombination between members of two distinct classes of DNA viruses has opened the gates to a new field of human disease development. In all cases studied thus far interviral recombination is a rare event that requires special circumstances for intracellular interaction of participating viral genomes. The rarity and special requirements do not detract from the potential clinical significance of resulting recombinants, as exemplified by recombination between JC viral and Epstein-Barr viral genomes. This significance depends largely upon the mechanisms of recombination that would generate specific forms of recombinant viral genomes. At this time little is known regarding mechanisms of interviral recombination. DNA break-induced replication seems presently to be a highly plausible means of initiating formation of different, potentially active recombination products. Generalizing interviral recombination to a variety of viruses will open a fertile field for discovery as multiple diseases of mysterious etiology are investigated.

## Introduction

At first consideration the observation that the distinct human viruses, JC virus (JCV) and Epstein-Barr virus (EBV) can undergo interviral recombination [1] may seem surprising. Previous results, however, have led up to this finding [2], and the replication properties of these two viruses provide special circumstances for their interaction. Recombination between human viruses has been frequently documented, generally involving two viruses of the same class or two distinct but closely related viruses, such as HIV-1 and HIV-2 [3]. In all cases reported thus far, interviral recombination is a rare event. As in these earlier documented cases, JCV and EBV share several special characteristics fostering recombination. There is ample evidence that both DNA viruses can co-infect certain cell types, that they can replicate in circular form during S-phase of the cell cycle, and that they utilize the same DNA polymerase replication apparatus in the same cellular compartments. These cumulative special conditions for JCV-EBV recombination raise the question of how extensively the significance of interviral recombination applies to human DNA viruses in general.

## Relation of Significance of Interviral Recombination to Underlying Mechanisms

**Figure 1:**
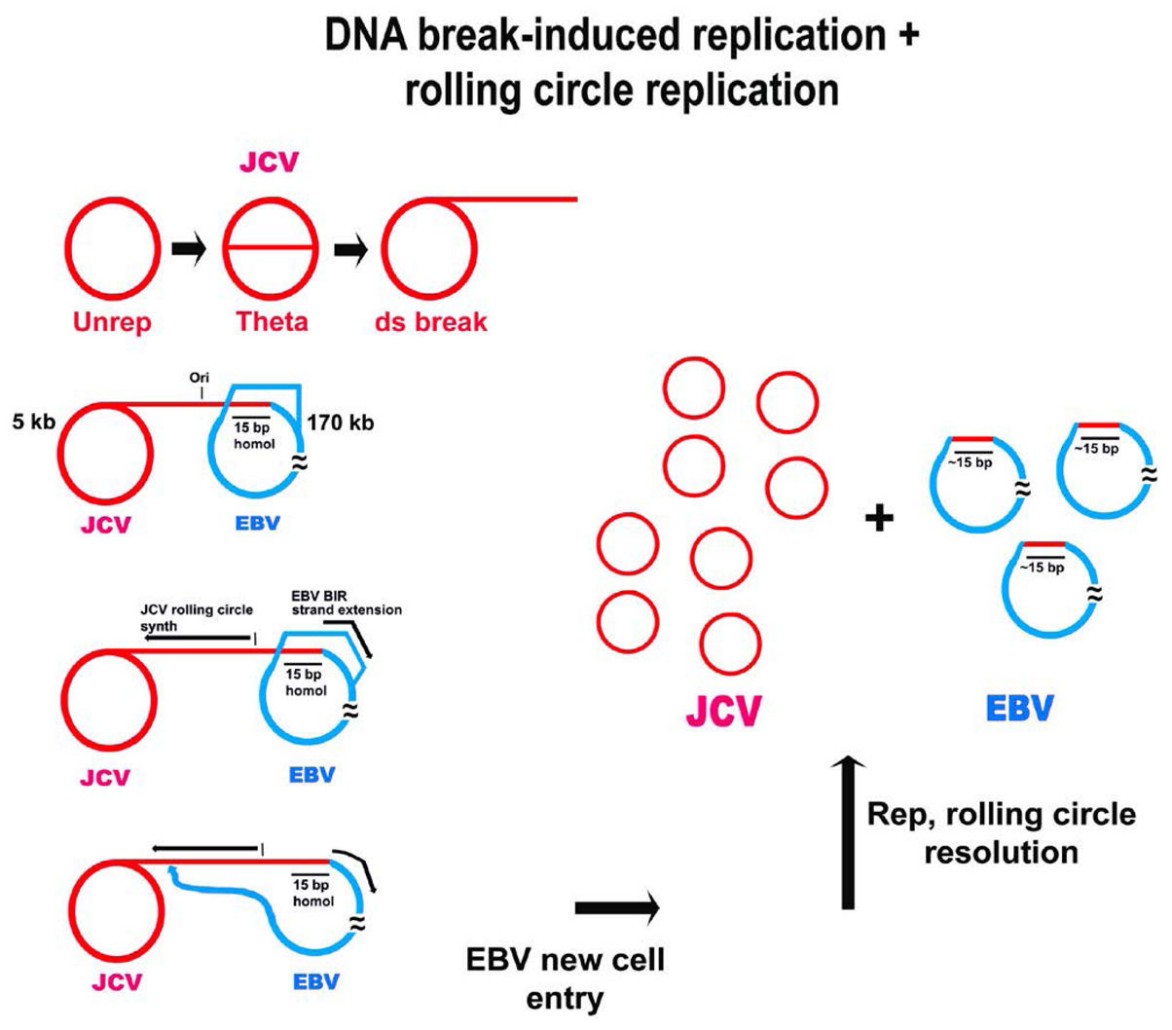
Mechanism of interviral recombination between JCV and EBV involving DNA break-induced replication (BIR) and rolling circle replication. **Note:** In this example, JCV inserts a 3′ strand end, resulting from a broken replication form (Theta), into the site of a demonstrated 15 bp homology in EBV. BIR is initiated in one direction in EBV. The other 3′ end in JCV continues DNA synthesis as a rolling circle. Completion of an EBV episomal circle may involve strand insertion at the same 15 bp homology, or it may involve non-homologous end joining at a broken, stalled JCV replication fork. Rolling circle resolution is standard to polyomaviruses. Copies of the resolved JCV genome may possess a deletion of approximately 15 bp at the homology, whereas EBV may acquire an insertion of this sequence.

Assessment of important aspects of the clinical significance of interviral recombination requires understanding of the mechanisms underlying this process. Elucidating such recombination mechanisms will address three major questions concerning significance. First, when two DNA viruses recombine, can one virus incorporate the complete genome of the second virus? This is of particular importance regarding circular viruses, and can be illustrated using the example of JCV and EBV. JCV is a small polyomavirus, with double-stranded DNA of approximately 5 kb, that replicates in circular form initiated from a single origin of replication located in its control region (NCCR). EBV is a very large gamma-herpes virus of about 180 kb that can replicate as circular episomes initiated from its latent ori P. A speculative model describing the structural interaction of these two viral genomes is presented in Figure 1. In this model JCV incorporated into EBV, perhaps with deletion of EBV sequences, could in an accommodating cell, initiate DNA synthesis, conceivably resulting in products including intact JCV molecules. In this scenario one virus, EBV, can carry a second viral genome, that of JCV, into a new cell type. A recombination model of this sort can help explain how JCV enters oligodendrocytes; this raises the second major question. Can the genome of one virus, say JCV, incorporate major pieces of the genome of another virus, say EBV in such a way that JCV can capitalize on the ability of EBV DNA to pass from one cell type to another in the absence of viral particles [4]? As a corollary of this question, can the gene expression of DNA segments of one virus be altered by incorporation into a second viral genome? Third, can recombination between two viral genomes, or segments thereof, form a hybrid genome with properties distinct from those of either virus alone? This could have implications for the ultimate generation of new viral diseases.

## Molecular Mechanisms of Interviral DNA Recombination: Potential Importance of Break-Induced Replication

A plausible mechanism that would explain all aspects of the type of JCV-EBV recombination thus far observed has yet to be proposed or tested. There are three sequences in the JCV NCCR that bear significant homology to any sequences in the entire EBV genome. These homologies are all at sites in the NCCR that are rearranged in the forms of JCV found in the demyelinating disease, PML [1]. These homologies do not readily suggest participation in any known model of recombination. All of the homologies are relatively short, the longest being 15 bp. Although possible, it is not clear that a homology of that length would easily initiate homologous recombination, a model that could explain some of the rearrangements seen in PML. We have documented a recombination product or intermediate in which a sequence from JCV leads into an EBV sequence, switching occurring at the 15 bp homology. One type of recombination that could account for such a hybrid sequence product is based on DNA break induced replication (BIR). In this model the 3′ end of a strand produced by a double-stranded DNA break inserts itself into a different double-stranded molecule at a site of a microhomology [5,6]. A 15 bp homology may suffice as such a small homology. Ensuing DNA synthesis could then generate the type of JCV-EBV hybrid product observed (Figure 1). It is not yet clear how initiation of this type of recombination would result in incorporation of one genomic segment into the other genome, but one plausible mechanism has been illustrated [5]. It is also conceivable that a composite mechanism takes place involving more than one known recombination model. A speculative model for JCV-EBV recombination initiation by BIR has been put forth [1] and expanded in Figure 1. Here it is interesting that BIR utilizes at least one protein not usually required for DNA replication [7]. Such proteins could be targets for a biomarker or for therapy.

## Clinical Significance of Generalized Interviral Recombination

**Figure 2:**
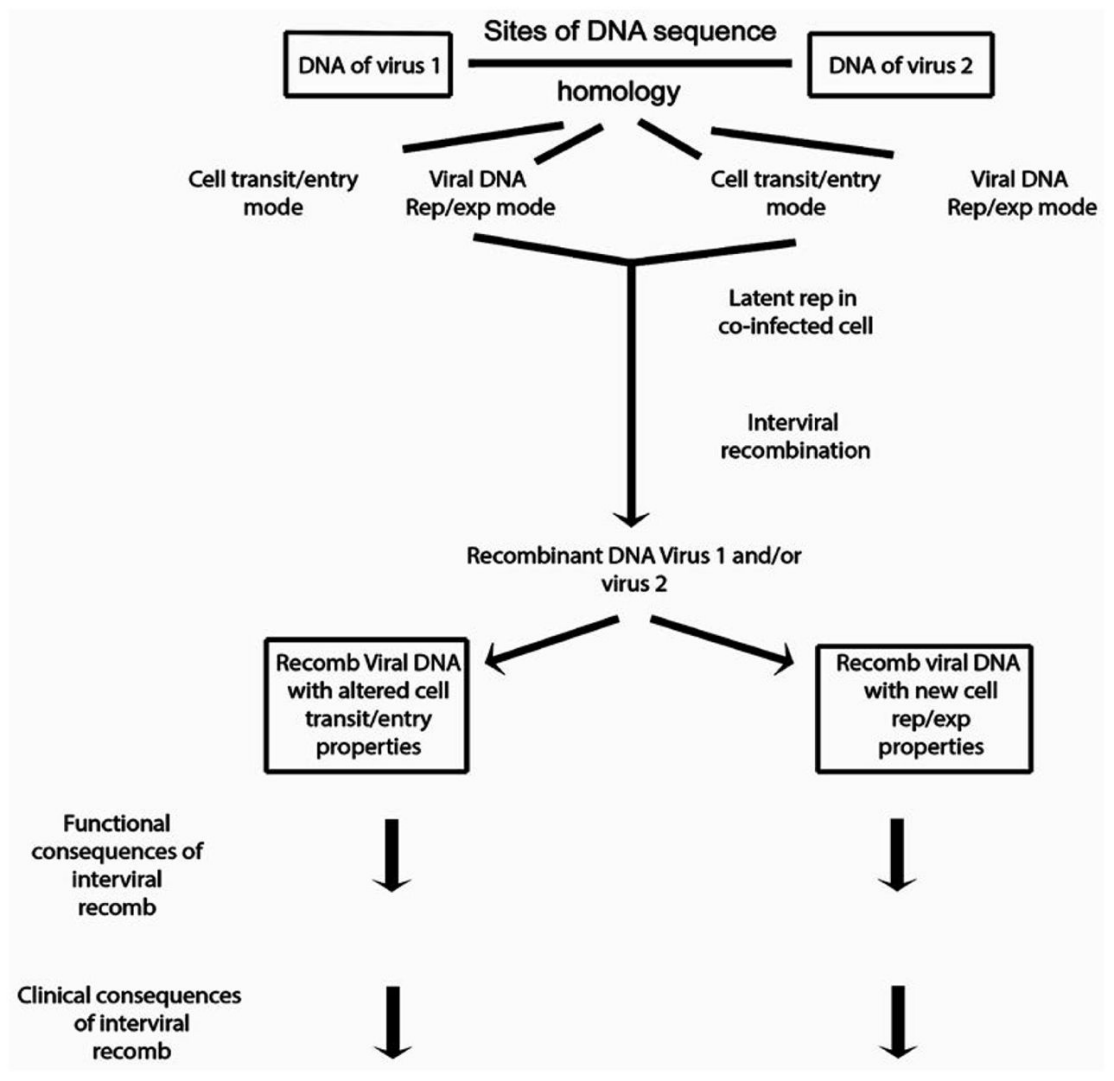
Order of events in interviral recombination leading to functional and clinical consequences. Lines without arrowheads=pointers to states of objects or modes. **Note:** Lines with arrowheads=order of timing of events. Rep=replication. Exp=expression. Recomb=recombination. Virus 1=an exogenous DNA virus or a DNA integrated form of an RNA virus. Virus 2=another virus in DNA form, and of a class distinct from virus 1.

**Figure 3:**
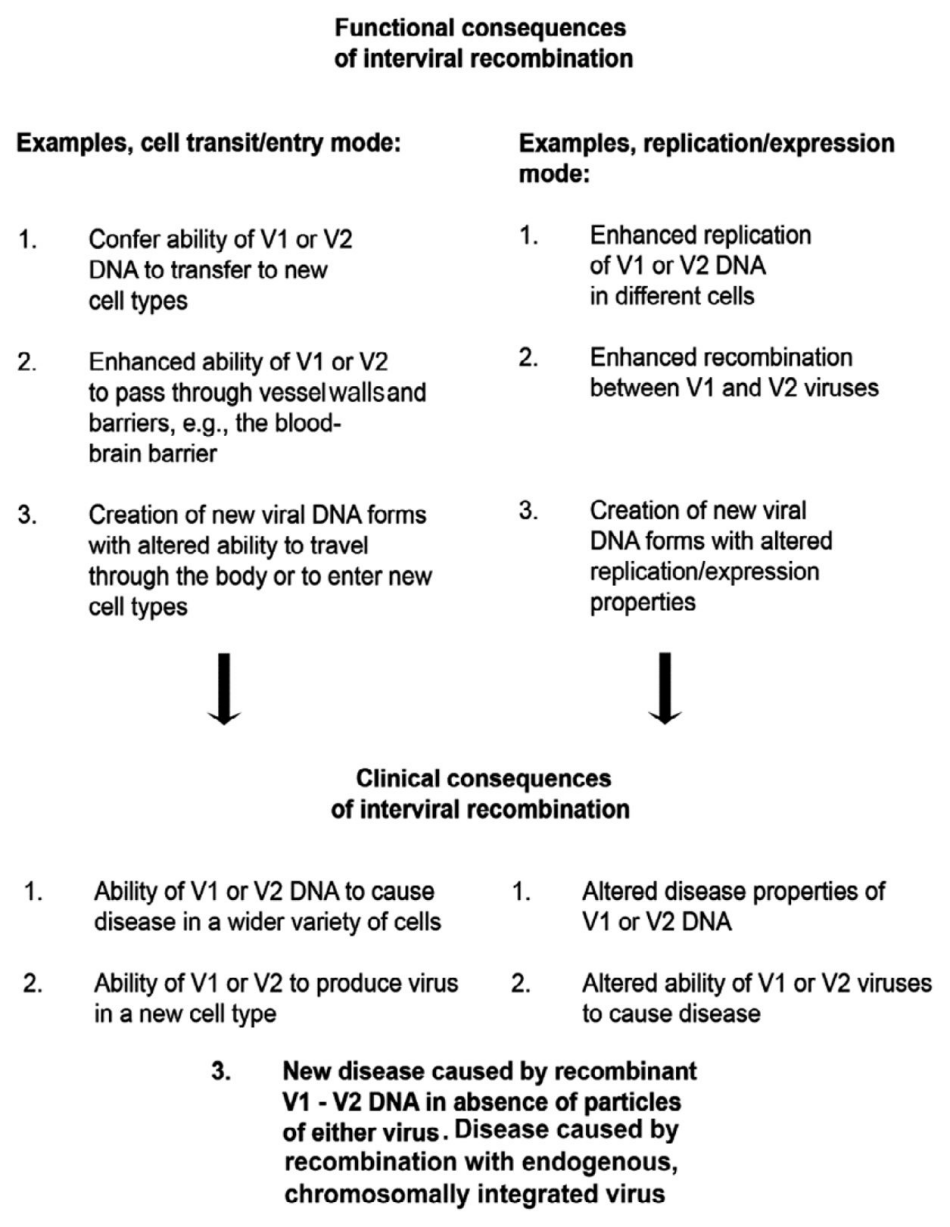
Functional consequences leading to clinical consequences of interviral recombination. The consequences of the two comprehensive modes of changes in viral properties are presented.

As one considers interviral recombination in general, certain of the clinical implications described for JCV-EBV recombination are further clarified. One can use JCV-EBV interviral recombination as an example to generalize such recombination between different human viruses. As discussed, JCV-EBV recombination requires special circumstances, and it is likely that recombination among different viruses will require special circumstances as well. It is likely, therefore, that all or nearly all interviral recombination events are rare. Can such rare events be of great significance? They may be so. The JCV-EBV recombination described above involves latent EBV in which genes necessary for productive activation have been disabled [1]. We have thus discussed consequences of this recombination in terms of capabilities of recombined genomic DNA. Assume, however, that one viral genome, say a rearranged JCV, can be transferred as an EBV recombinant to a new cell type so that the JCV genome is either intact or capable of reconstitution. Assume also that the new cell type can make JCV virus particles. In that case any new disease caused by JCV in the new cell type may be self-propagating although still rare. This example could help explain how JCV can transit from its known peripheral body tissues to enter the brain and infect oligodendroglial cells. Note that in these circumstances recombination may continue in the oligodendroglia. Alterations of two modes of function are important in order to achieve consequences that extend to clinical significance for JCV-EBV interviral recombination. Those are a transit/entry mode and a replication/expression mode. Recombination altering the ability of one or both viral genomes to travel between tissues or cell types and to enter a new cell type is important. In addition, alteration of the ability of a viral genome to replicate and to express its specific genes is important. Although there may be many different viral genomic loci critically altered to achieve significance for interviral recombination, all resulting changes can be roughly grouped under these two functional modes. Using the transit/entry mode and replication/expression mode to classify viral genomic alterations, we present in Figures 2 and 3 a generalized route to the clinical consequences of interviral recombination.

## Potential Role of Interviral Recombination in Various Diseases

There are several diseases, the etiology of which may now be assessed in terms of interviral recombination. Recombination between HIV-1 and HIV-2 has been demonstrated after coinfection with genetically modified viruses and has not thus far been shown in an infected population. Although its consequences cannot be assessed, they are potentially intriguing because each virus may influence cell entry of the other and because these viruses differ in propensities for pathology in AIDS. JCV-EBV recombination has been shown in immunosuppressed individuals [1], and is presently open for assessment of consequences. PML may be the most direct example of such a consequence because it involves JCV with sequence alterations at the sites of potential recombination with EBV, as noted above. JCV does not infect the brain in people without PML. The virus causes a low-level, asymptomatic infection of uroepithelial tissue in most people as well as a low-level or latent infection of bone marrow and tonsillar tissues, including B cells that can also harbor EBV [8]. JCV must transit from its peripheral sites to the brain, where it infects oligodendroglia to cause demyelination. EBV can infect multiple epithelial cells and B cells, where it may influence the transit of JCV via interviral recombination as described above. Another disease to consider with regard to recombination involving EBV is multiple sclerosis (MS). MS is a demyelinating disease affecting oligodendrocytes. Although PML and MS are similar in that respect, they are distinct in pathology, and MS progresses to debilitation much more slowly than does PML. EBV is very strongly linked serologically to MS [9], but neither EBV nor JCV is found in MS lesions. It may be that a recombination form of EBV is etiologically linked to MS, and that form may not persist in all stages of the disease. Note here that both EBV and JCV can recombine with or integrate into chromosomal human genomic DNA. Genomic DNA contains many endogenous retroviruses (HERVs), presumed to be of ancient origin and inactive. It is possible that recombination with an exogenous virus such as EBV or JCV can activate a dormant HERV. This would be a special case of Clinical consequences, No. 3, in Figure 3: a new disease caused by recombination of V1 or V2 DNA with genomic DNA in the absence of virus particles. There is a strong possibility that HERV activation involves recombination. It is an attractive notion that this involves an exogenous virus, recombining either directly with the HERV or with sequences adjacent to its integrated form. It is clear that the demonstration of interviral recombination among human DNA viruses has pointed to only the most readily identifiable instances of significance, and that this will be a fertile field for discovery as more diseases of mysterious etiology are examined.

## References

[1] Wortman MJ, Lundberg PS, Dagdanova AV, Venkataraman P, Daniel DC (2016). Opportunistic DNA Recombination With Epstein-Barr Virus at Sites of Control Region Rearrangements Mediating JC Virus Neurovirulence. J Infect Dis.

[2] Johnson EM, Wortman MJ, Lundberg PS, Daniel DC (2015). Orderly steps in progression of JC virus to virulence in the brain. Brain Disorders & Therapy.

[3] Motomura K, Chen J, Hu WS (2008). Genetic recombination between human immunodeficiency virus type 1 (HIV-1) and HIV-2, two distinct human lentiviruses. J Virol.

[4] Nanbo A, Terada H, Kachi K, Takada K, Matsuda T (2012). Roles of cell signaling pathways in cell-to-cell contact-mediated Epstein-Barr virus transmission. J Virol.

[5] Hastings PJ, Ira G, Lupski JR (2009). A microhomology-mediated break-induced replication model for the origin of human copy number variation. PLoS Genet.

[6] Sakofsky CJ, Ayyar S, Deem AK, Chung WH, Ira G (2015). Translesion Polymerases Drive Microhomology-Mediated Break-Induced Replication Leading to Complex Chromosomal Rearrangements. Mol Cell.

[7] Lydeard JR, Lipkin-Moore Z, Sheu YJ, Stillman B, Burgers PM (2010). Break-induced replication requires all essential DNA replication factors except those specific for pre-RC assembly. Genes Dev.

[8] Houff SA, Berger J (2010). The curious incident of the dog in the nighttime: does the absence of virus replication in Epstein-Barr virus-transformed B cells point to an important feature of JC virus biology?. J Infect Dis.

[9] Goodin DS (2009). The causal cascade to multiple sclerosis: a model for MS pathogenesis. PLoS One.

